# 

**Published:** 1887-09

**Authors:** 


### EDITORIAL.

### The Ohio State Dental Society.

The next annual meeting of this body will be held in Springfield on Wednesday, October 26, and continue three days. A full programme will be published in the next number of the REGISTER. Attention is now called to this meeting in order that members and visitors may not only have the matter in mind, but make arrangements to be present; and have time to make preparations, to add to the work, interest, and profit of the occasion.

It is to be hoped, that so far as possible, every one who may attend will be prepared to say something, do something, or exhibit something that will benefit others, and thus give while he receives blessing.

Effort is being made to have this the largest and best meeting of this society ever held, and we trust that this effort will be answered to the full. This is one of the primitive State Societies, and its experience warrants a high expectation as to work accomplished; and let every member see to it that there is no disappointment. Let every dentist in the State who has any regard for and appreciation of his profession make it a point to be present at this meeting. Springfield is a very accessible and central point.

### New England Dental Society.

The twenty-fifth anniversary meeting of the New England Dental Society will be held in Boston Wednesday, Thursday, and Friday, October 5th, 6th, and 7th.

It is intended at this time to have the work of the meeting appropriate to the occasion. It is very desirable that all the members, and as many visitors as possible from all quarters, be present.

There will be presented a number of good papers. Arrangements have been made for good clinics and the exhibition of new instruments and appliances. No one within reach can afford to remain away from this meeting. Each and every one should add something to the pleasure and profit of the occasion. In order that proper arrangements may be made, every one should notify Dr. A. M. Dudley, Secretary, Salem, Mass., at the earliest possible moment.

May this be the largest, most interesting, and profitable meeting of this body ever held.

### THE DENTAL REGISTER,

A MONTHLY MAGAZINE DEVOTED TO THE INTERESTS OF THE

**DENTAL PROFESSION.**

*Terms Reduced to* $2.00 *Per Year. Single Copies,* 25 *Cents.*

**EDITED BY PROF. J. TAFT, D.D.S.**

—AND—

**Published by SAM'L A. CROCKER & CO., Ohio Dental and Surgical Depot,**

The volume commences in January and closes in December.

The REGISTER being issued monthly is always fresh, therefore Indispensable to the practitioner who desires to keep posted up with the new inventions and improvements which are daily developed. Neither expense nor effort will be spared to give value and variety to its contents. Articles will be illustrated with well executed engravings whenever they will add to the interest or assist to explanation.

Its extensive circulation and frequency of issue make it one of the BEST DENTAL ADVERTISING MEDIUMS in the United States.

All subscriptions must begin with January or July.

Local or special notices, five lines or less, will be inserted for $3 each insertion ; over five lines, 50 cts. per line.

All advertising bills payable in advance, or at furthest, after the issue of the first number containing the advertisement. All transient advertisements to be paid for in advance.

☞CONTRIBUTIONS SOLICITED.

Exclusively Editorial Communications should be addressed to the Editor.

The latest number of the REGISTER may be ordered with or without other goods at any time, and all letters should be addressed to

SAM'L A. CROCKER & CO., Ohio Dental and Surgical Depot, Cincinnati, O.

